# Effects of Cannabis Use on Human Brain Structure in Psychosis: A Systematic Review Combining *In Vivo* Structural Neuroimaging and *Post Mortem* Studies

**DOI:** 10.2174/138161212802884861

**Published:** 2012-11

**Authors:** Charlotte Rapp, Hilal Bugra, Anita Riecher-Rössler, Corinne Tamagni, Stefan Borgwardt

**Affiliations:** 1 Department of Psychiatry, University of Basel, 4031 Basel, Switzerland; 2 Medical Image Analysis Centre, University of Basel, Switzerland; 3 King’s College London, Department of Psychosis Studies, De Crespigny Park, London SE5 8AF, United Kingdom

**Keywords:** Cannabis, post-mortem, neuroimaging, At-risk mental state (ARMS), psychosis, magnetic resonance imaging (MRI)

## Abstract

It is unclear yet whether cannabis use is a moderating or causal factor contributing to grey matter alterations in schizophrenia and the development of psychotic symptoms. We therefore systematically reviewed structural brain imaging and post mortem studies addressing the effects of cannabis use on brain structure in psychosis. Studies with schizophrenia (SCZ) and first episode psychosis (FEP) patients as well as individuals at genetic (GHR) or clinical high risk for psychosis (ARMS) were included. We identified 15 structural magnetic resonance imaging (MRI) (12 cross sectional / 3 longitudinal) and 4 post mortem studies. The total number of subjects encompassed 601 schizophrenia or first episode psychosis patients, 255 individuals at clinical or genetic high risk for psychosis and 397 healthy controls. We found evidence for consistent brain structural abnormalities in cannabinoid 1 (CB1) receptor enhanced brain areas as the cingulate and prefrontal cortices and the cerebellum. As these effects have not consistently been reported in studies examining non-psychotic and healthy samples, psychosis patients and subjects at risk for psychosis might be particularly vulnerable to brain volume loss due to cannabis exposure

## 1 INTRODUCTION

Cannabis is the world’s most widely used illicit drug with about 10% of young adults in developed countries being regular users. Behavioural and pharmacological studies indicate that both acute and chronic exposure to cannabinoids is associated with impairments in a range of cognitive processes [1-7]. Neuroimaging methods have provided powerful tools to study the *in vivo *effects of cannabis on brain function. While there are brain functional differences, such as reduced resting-state, prefrontal and anterior cingulate cortex blood flow, between cannabis users and controls in healthy populations, brain structural abnormalities related to cannabis use have been reported inconsistently [8-10]. In contrast, a neurotoxic effect (e.g. shrinkage of neuronal cell bodies and nuclei) of cannabis in animals has been confirmed in many cases [11, 12]. It is assumed, that Δ9-tetrahydrocannabinol (THC), the main psychoactive substance in cannabis, is the neurotoxic substance [13]. The effects of cannabis on brain structure and function are of particular interest in psychosis patients, as cannabis is known to be a risk factor for psychosis [14-16] and is widely used in patients with psychosis [17]. There is evidence from structural imaging studies showing robust volume reductions in fronto-temporal cortices and in the anterior cingulate in patients with schizophrenia [18-37] suggesting that these changes are underlying pathophysiological processes of this disorder. Cannabis use may therefore be a moderating or causal factor contributing to grey matter alterations in schizophrenia and the development of psychotic symptoms. 

De Lisi [38] reviewed evidence to suggest that there are detectable brain changes occurring as a consequence of cannabis use that lead to increased risk of psychosis. It was concluded that this is unlikely as cannabis might even have protective effects on brain structure and not produce deleterious damage. However, only four MRI studies of people with schizophrenia who used cannabis had been considered in this review. In the meantime, many more MRI studies have been published to this subject. This review therefore systematically reviewed structural neuroimaging studies addressing the effects of cannabis use on brain structure in psychosis. In addition, findings from *post mortem* studies investigating the effect of cannabis on cannabinoid receptor density were included. In order to contribute to the question whether cannabis use is associated with structural brain abnormalities during development of psychosis, subjects at high clinical risk and with an at risk mental state (ARMS) as well as genetic high-risk (GHR) individuals were included. 

## 2 METHODS

### 2.1 Selection strategy

#### 2.1.1 Search Strategy

Electronic searches were performed using ISI Web of Knowledge and PUBMED database. We included all studies published until end of November 2011 without any language restriction, according to well defined inclusion criteria - see below here. The following key words were used: “psychosis”, “schizophrenia”, “first episode”, “at-risk mental state”, “high risk”, combined with “cannabis”, “marijuana”, “delta-9-tetrahydro-cannabinol” (THC), and “brain structure”, “neuroimaging”, “brain imaging”, “brain abnormalities”, “magnetic resonance” (MRI), “diffusion tensor MRI” (DTI), “post mortem”, “quantitative autoradiography”, “radioligand binding”, “in situ hybridization”. Patients met diagnostic criteria for schizophrenia, schizophreniform or schizoaffective disorder according to Diagnostic and Statistical Manual of Mental Disorders DSM-III-R or DSM-IV criteria. Subjects at risk for psychosis fulfilled the At Risk Mental State (ARMS) criteria [39] or were at familial risk for schizophrenia (siblings [40] / at least two members of family from subject suffer from schizophrenia [41, 42]). We also carefully searched the reference lists of the included articles identified in the original search for further relevant articles.

#### 2.1.2 Selection Criteria

We initially performed a general review of all studies investigating brain structure of patients (established schizophrenia, individuals at clinical risk for psychosis and individuals at genetic risk for psychosis) in relation to cannabis use. Studies were only included if they met the following criteria: (a) be an original publication in a peer-reviewed journal (b) studying the brain of psychosis patients (first episode, FEP or chronic schizophrenia, SCZ) or individuals at risk for psychosis (ARMS) or individuals at genetic risk for psychosis (GHR) in relation to cannabis use, applying *in vivo* structural neuroimaging or *post mortem* autoradiography or in situ hybridization techniques (c) including both cannabis smokers and non-smokers (d) extracting the specific effects of cannabis on brain if subjects had a general substance abuse or substance dependence disorder diagnosis. As this review was focused on brain structure, we only included structural imaging studies (MRI/DTI) investigating both gray and white matter. Functional brain imaging studies (e.g. fMRI, PET) were excluded. *Post mortem* brain studies allow localization of abnormalities in the endogenous cannabinoid system. We restricted the analyses to brain regions least subject to autolytic processes and on CB1 receptors given its central role in mediating endogenous cannabinoid function. To quantify changes in CB1 receptors in schizophrenia, the following methods have been used: 1) in situ radioligand binding and autoradiography and 2) in situ hybridization and immunocytochemistry.

We included all studies which involved cannabis using patients, regardless of whether they fulfilled the criteria for a substance use disorder or not. The amount of how much cannabis was consumed by the subjects varied widely across the studies. Although there were studies with overlapping samples [41-49], they analysed different brain regions or used cross sectional vs. longitudinal contrasts. 

### 2.2 Recorded Variables

Two of the authors extracted the data independently (CR and HB). When there was no agreement, a third author (SB) reviewed the paper independently. Results were reported in different tables to assist the reader in establishing an independent view on the topic. We have included two summary tables of all reviewed structural MRI (Table **1**) and *post mortem* studies (Table **2**), one table illustrating the *in vivo* effects of cannabis on brain imaging results (Table **3**) and one table presenting the *post mortem* results (Table **4**). The recorded variables for each article included in the review were: centre where the study was performed, authors and year of publication, main subject, study design, number of subjects overlapping with other studies, number of subjects, mean age, percentage males/females, instrument for cannabis use assessment, definition of cannabis use, image analysis method, regions and structures of interest. The primary outcome measures of interest for MRI studies were global and regional gray and white matter volumes/density as well as density of cannabinoid receptor binding for the post mortem studies.

### 2.3 Risk of Bias in Individual Studies 

Publication bias expresses the higher probability of a study being published when it has a positive result [50] – thus, an intrinsic bias towards a positive result could be incorporated into a review study. From the studies included in this review, 12 studies did find a structural difference between cannabis users and non-users and 7 did not find one. All the included studies were published in peer-review journals suggesting high quality of data and methodology. We did not find differences in outcome-level assessment of risk bias.

## 3 RESULTS

### 3.1 Identified Studies

**Fig. 1. Flow diagram (selection strategy) of included studies:**
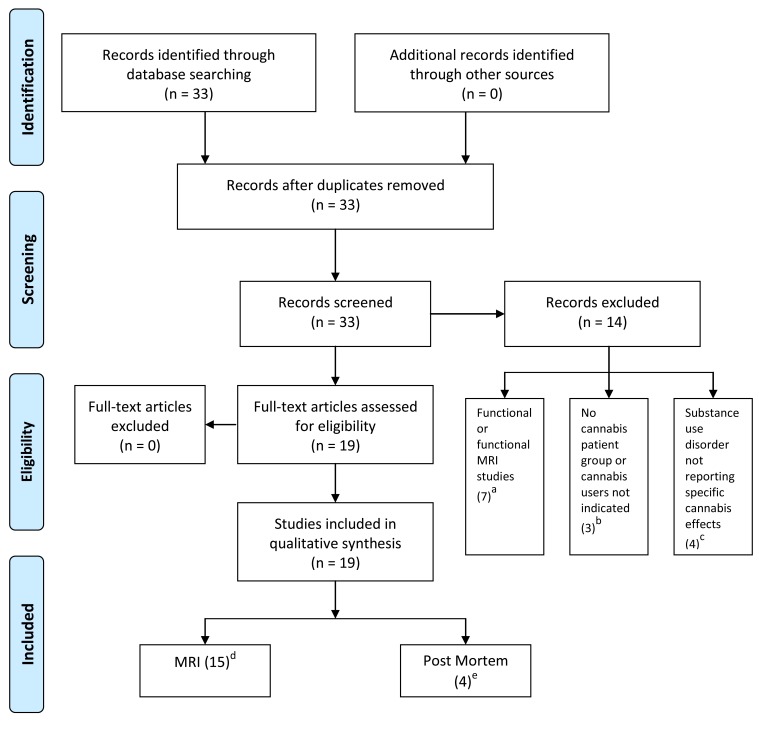
^a^ Mancini-Marie *et al*. 2006 [76]; Jockers-Scherübl *et al*. 2003 [68]; Leweke *et al*. 2007 [77]; Safont *et al*. 2011 [78]; Wobrock *et al*. 2010 [88]; Loberg *et al*. (2011) [79]; Potvin *et al*. 2007 [80]. ^b^ Parkar *et al*. 2001 [81]; Newell *et al*. 2006 [82]. Dalton *et al*. 2011 [83]^c^ Potvin *et al*. 2007 [84]; Wobrock *et al*. 2009 [85]; Ebdrup *et al*. 2010 [86]; Koethe *et al*. 2006 [87]. ^d^ Habets *et al*. 2011[40]; Ho *et al*. 2011[56]; James *et al*. 2011[57]; Cohen *et al*. 2011[53]; Solowij *et al*. 2011 [52]; Stone *et al*. 2011[39]; Welch *et al*. 2011[41]; Welch *et al*. 2011[42]; Dekker *et al*. 2011[46]; Peters *et al*. 2009[47]; Rais *et al*. 2010[44]; Rais *et al*. 2008 [43]; Cahn *et al*. 2008 [45]; Bangalore *et al*. 2008 [55]; Szeszko *et al*. 2007 [54]. ^e^ Eggan *et al*. 2008 [60]; Zavitsanou *et al*. 2004 [49]; Dean *et al*. 2001 [59]; Deng *et al*. 2007 [48]

All included studies were published between 2001 and 2011, whereby 9 (out of 19) were published in 2011. Out of 33 initially screened studies, 13 were excluded because they did not fulfil the inclusion criteria. Functional studies were not considered in this review because we aimed to look for effects of cannabis on brain structure. The flowchart of the selection procedure with the included/excluded studies is summarized in (Fig. **1**) and was based on the template of the PRISMA flow diagram [51] available on www.prisma-statement.org. For included and excluded studies see (Fig. **1**). The remaining studies were grouped according to centre/population of the study, method (sMRI, post mortem autoradiography) and study design (cross sectional vs. longitudinal) (Fig. **1**, Table **1** and **2**). The systematic review of the literature uncovered 15 in-vivo structural gray/white matter MRI/DTI studies and 4 post mortem studies (three autoradiography and one in situ hybridization study). The total number of subjects included in this review encompassed 601 FEP / SCZ (mean age = 27.05 years, age range 16.3 – 47.9, 20.4% females) (of which around 280 were cannabis users), 255 ARMS / GHR (mean age = 23.8 years, age range 21.16-29.5, 47% females) (around 160 cannabis users) and 397 healthy controls (HC) (mean age = 28.17 years, age range: 16.4-48.0, 30% females (around 70 cannabis users). 

Within our included studies we did not find any differences in risk of bias. In the following, the results of our systematic review are summarized with respect to *in vivo* (section 3.2.) and post mortem (section 3.3.) studies.

### 3.2 *In Vivo* Structural Imaging results

The results of all *in vivo* studies (n = 15) are specified in Table **3**.

#### 3.2.1 Cross Sectional Structural Imaging Studies

Twelve studies have investigated cross-sectionally how cannabis affects brain structure in psychosis. Eight have looked at established psychosis and included SCZ or FEP (section 3.2.1.1). Four studies included subjects at high-risk for psychosis with 228 GHR and 54 ARMS subjects (3.2.1.2). 

##### 3.2.1.1 Cross Sectional Structural Imaging Studies in Established Adult-onset Psychosis

Three studies focused on cerebellar changes in cannabis users. Solowij *et al*. [52] examined cerebellar grey and white matter in cannabis users (C+) and non-users (C-) with and without chronic schizophrenia. They found that cerebellar white matter in healthy cannabis users (C+ HC) was 23.9% and 29.7% smaller in schizophrenic cannabis users (C+ SCZ) than in non-using healthy controls (C- HC). As the difference in cerebellar white matter volume between schizophrenia patients who did not use cannabis (C- SCZ) and healthy controls (C- HC) was 17.7%, the authors concluded that cannabis might have a greater adverse effect on white matter than the effect of schizophrenia. Another recently published study [53] of a FEP sample found that cannabis use was associated with reduced cerebellar grey matter volume in a dose-dependent matter in C+ HC. However, in FEP, there was neither an effect of cannabis use, nor an interaction between cannabis use and diagnosis on cerebellar grey matter. Cahn *et al*. [45] compared total brain volumes, cerebral, cerebellar, caudate, lateral and third ventricle volumes between recent onset schizophrenic patients with a comorbid DSM IV cannabis abuse/dependence diagnosis (C+ SCZ) and without (C- SCZ). No differences in these brain regions of interest between C+ SCZ and C- SCZ were found.

Szeszko *et al*. [54] investigated the superior frontal gyrus, anterior cingulate gyrus and the orbital frontal lobe in a sample of first episode psychosis patients with and without DSM IV cannabis use/dependence disorder (C+ FEP / C- FEP) and a non-consuming healthy control sample (C- HC). They found that C+ FEP had significantly less anterior cingulate grey matter than C- FEP and C- HC. This finding could be replicated in a similar study [55] in the posterior cingulate cortex, which reported that C+ FEP had significantly less right posterior cingulate cortex and less left hippocampal volume than C- FEP. Further analyses in this study of C+ FEP versus C- HC showed a trend for a decrease in the right posterior cingulate grey matter. No differences were noted between C- FEP and C- HC. 

A recent study by Ho *et al*. [56] examined the effect of cannabinoid receptor 1 (CB1) gene polymorphisms and cannabis use on brain structure in a sample of SCZ. The hypothesis was that patients with specific CB1 genotypes would be more vulnerable to the damaging effects of cannabis abuse regarding to brain volume. 

C+ SCZ had smaller frontal white matter than C- SCZ. Grey matter, parietal white matter and lateral ventricle volumes did not differ between the two groups. 

##### 3.2.1.2 Cross-sectional Imaging Studies in Psychotic Psychosis Subjects During Adolescence

James *et al.* [57] examined the effects of cannabis use during adolescence in a sample of adolescent onset SCZ [57]. All subjects were aged between 13 and 18 years. The hypotheses were that the effects of chronic cannabis use in schizophrenia would be particularly severe during adolescence critically involved in neurodevelopmental processes. The results showed that C+ SCZ had reduced grey matter in temporal fusiform gyrus, parahippocampal gyrus, ventral striatum, right middle temporal gyrus, insular cortex, precuneus, right paracingulate gyrus, dorsolateral prefrontal cortex, left postcentral gyrus, lateral occipital cortex and cerebellum. They also showed decreased fractional anisotropy (FA) in brain stem, internal capsule, corona radiate, superior and inferior longitudinal fasciculus compared to C- SCZ.

Two DTI studies from Amsterdam [46, 47] assessed retrospectively whether heavy cannabis use occurred before age 17 or not. The first earlier study [47] compared recent onset SCZ aged around 22 years with cannabis use before age 17 versus patients without cannabis use before age 17 versus C- HC. Fractional anisotropy in the anterior internal capsule, fasciculus uncinatus and frontal white matter was higher in C+ SCZ before age 17 compared to C- HC. There was no significant difference between C- HC and C- SCZ before age 17. However, most C- SCZ before age 17 also did not smoke cannabis after. Therefore, it could not be excluded that the results were due to the cannabis effect in general rather than to critical use during adolescence. The later study from the same centre [46] showed reduced white matter density in the left posterior 

corpus callosum, right occipital and left temporal lobe in C- SCZ compared to early onset C+ SCZ. 

##### 3.2.1.3 Cross-sectional Structural Imaging Studies in Subjects at Clinical or Genetic Risk for Psychosis

Four studies examined the effect of cannabis use on brain morphology in subjects at risk for psychosis with three studies of subjects at genetic high risk for psychosis (GHR) and one study of subjects with an at risk mental state (ARMS) [58] sample. The results from a longitudinal study with GHR subjects will be presented later [42].

A prospective cohort study with case control comparison design [41] analysed the association between substance misuse (alcohol and cannabis), brain morphology and subsequent schizophrenia in GHR subjects. Correlational analyses showed significant negative dose-dependent associations between cannabis use and lateral and third ventricle sizes. These associations were absent in the control group. Additionally, those GHR subjects with at least regular use of cannabis had a higher risk of later developing schizophrenia than those with isolated or no use.

Another study with GHR subjects was conducted by Habets *et al*. [40] who included C+ and C- of three groups: SCZ, GHR and HC. They found a significant group × cannabis interaction on cortical thickness, indicating that the effect of cannabis varied as a function of group. C+ SCZ had significantly lower cortical thickness values than C- SCZ. This pattern was similar in GHR but not in HC.

The only study with ARMS subjects [39] reported a negative correlation between cannabis intake and grey matter volume in prefrontal cortex, cingulate and left insula. However, there was no difference between ARMS and HC, suggesting no specific susceptibility to the effects of cannabis on brain structure in ARMS.

#### 3.2.2 Longitudinal Structural Imaging Studies

Three longitudinal studies examined the effect of cannabis use over time on brain structure in psychosis patients and GHR individuals. Rais *et al*. [43] found that C+ FEP, C- FEP and C- HC did not differ with regards to global brain volumes at baseline. However, at follow up after 5 years, cannabis using patients (C+ FEP) showed larger gray matter volume loss and larger lateral and third ventricles than patients who did not consume cannabis during the scan interval (C- FEP) and compared to C- HC. This group [44] similarly reported no differences between the three groups at baseline but progressive regional density reduction during follow-up in the right supplementary cortex, left anterior cingulate cortex and left occipital lobe in FEP relative to HC. Patients who used cannabis during that time (C+ FEP) showed additional density reduction in the dorsolateral prefrontal cortex, left anterior cingulate cortex and left occipital lobe compared to C- FEP. 

Welch *et al*. [42] compared the thalamus and amygdala-hippocampus complex in GHR individuals with cannabis use during a scan interval of two years with high risk subjects who did not use cannabis during this period of time. At baseline, C+ GHR and C- GHR did not differ with regards to whole brain, thalamic or amygdala-hippocampal complex volumes. Cannabis exposure over time was associated with bilateral thalamic volume loss which was highly significant on the right side.

### 3.3 Post Mortem Results

Three studies investigated cannabinoid receptor binding in brains of SCZ at death and additionally reported the effects on the receptors caused through the use of cannabis. For an overview on the results of the studies refer to Table **4**. 

Dean *et al*. 2001 [59] used in situ radioligand binding and autoradiography to measure the binding of [^3^H]CP-55940 to the cannabinoid-1 receptor in the dorsolateral prefrontal cortex, caudate-putamen and areas of the temporal lobe from schizophrenic and control subjects. Five out of the 14 SCZ and four out of the 14 HC had a history of cannabis use and THC in their blood at death. SCZ showed an increase in the density of [^3^H]CP-55940 binding in the dorsolateral prefrontal cortext compared to HC. A significant increase in the density of [^3^H]CP-55940 binding in tissue was noted in the subjects who had THC in the blood at death. However, there were no differences in binding between lifetime C+ SCZ and C- SCZ. The same techniques were used in another study [49] which measured the binding of [^3^H]SR141716A on anterior cingulate cortex, an antagonist that specifically targets CB1 receptors. A significant increase in density of CB1 receptors receptors was found in SCZ compared to HC. No differences were noted in CB1 binding between C+ SCZ and C- SCZ. A later study from the same group [48] investigated binding densities of [^3^H]SR141716A and [^3^H]CP-55940 to the CB1 receptors in the superior temporal gyrus in the same sample. In contrast to the earlier results, no significant difference was found between SCZ and HC in receptor binding and there was also no effect of cannabis use. Another *post mortem* study [60] used in situ hybridization and immunocytochemistry techniques to measure the cortical levels of CB1 and protein in the dorsolateral prefrontal cortex in schizophrenic patients and controls. Levels of CB1R messenger RNA were significantly reduced in SCZ compared to HC but history of cannabis use did not account for any group differences.

## 4 DISCUSSION 

In this systematic review it was investigated whether cannabis use has an effect on brain morphology in psychosis patients and in subjects at clinical or genetic risk for psychosis. The present review focused on structural MRI and additionally reviewed *post mortem* studies examining brain structure based on cannabinoid receptor density. Our systematic search strategy and literature review uncovered consistent brain structural abnormalities in CB1 receptor-enhanced brain areas such as the cingulate, the prefrontal cortex and the cerebellum.

Few structural neuroimaging studies have investigated cannabis use in non-psychiatric populations yet and results were inconsistent. Limited evidence of major effects of cannabis on brain structure has so far been reported [8, 9] with the strongest effects being found in medial temporal regions [10]. We focused here on psychiatric patients affected with early or chronic phases of psychosis. Some methodological limitations must soon be acknowledged. For example, comparing results between studies presented in this systematic review is hindered by differences in subject selection and design of the studies. Not all studies used DSM-IV criteria for cannabis dependence or abuse and studies varied in how they set criteria to define their cannabis using (C+) and non-using (C-) group. A solution to overcome could have been to include cannabis as a continuous variable rather than creating dichotomous groups as it was done by a few studies in this review [40, 41]. However, the problem with most studies is also that cannabis intake is poorly measured across studies yielding to high between-samples heterogeneity. This point is very important because smoking styles and quantities vary largely and it has also been reported, that the THC content of smoked cannabis has increased over the past 20 years [61]. Another problem for quantitatively comparing the different included studies is the variety regarding the inclusion of a control group: some studies included a non-psychiatric sample and others did not. Within those studies that included a non-psychiatric sample, only few also analysed cannabis using healthy controls.

Despite these factors that make it difficult to compare the included studies at meta-analytical level, a few conclusions can be drawn. Within the 15 *in vivo* structural MRI studies included in this review, 11 found that cannabis use (as individually defined in each study) was associated with a decrease in global or specific brain structures in psychosis patients [40, 44, 52, 54-57, 62] or subjects at clinical/genetic risk for psychosis [39, 40, 42, 63]. These effects seemed to be particularly strong in brain regions rich on CB1 receptors, such as the cingulum [54, 55], the dorsolateral prefrontal cortex [44, 57] and the cerebellum [52, 57]. Two studies reported no difference between users and non users [45, 53] in psychosis and two studies reported that early onset cannabis users showed more white matter than cannabis naïve patients and controls [46, 47]. Conversely, none of the post mortem studies found an effect of cannabis use on cannabinoid receptor density except for Dean *et al.* [59] who reported an increase of CB1 receptors in the caudate-putamen from subjects who had recently ingested cannabis.

The effects of cannabis on the patient and control groups were differentially reported: while one study found that the effects of cannabis on brain structure are equally both in ARMS and controls [39], other studies showed that the negative effects of cannabis use on brain structure were significantly more pronounced in psychosis patients and genetic high risk subjects than in healthy controls [40, 41, 52]. In general, this review shows that effects of cannabis on brain in psychosis subjects seem to be more distinct than in studies investigating non-psychiatric samples [8, 9]. This could be an indication that people with schizophrenia or at genetic high risk for this disease may have a particular sensitivity to brain tissue loss on exposure to cannabis, which is also in line with previous research reporting that the use of alcohol and amphetamines is associated with greater structural brain abnormalities than this would be expected in healthy individuals with a comparable level of exposure [64, 65]. However, the question remains whether brain abnormalities are only an expected consequence of substance misuse or whether they also predispose it: cortical and hippocampal dysfunctions in schizophrenia could also be responsible for the greater reinforcement of drugs leading to more drug problems, which underlines the latter assumption [66].

### 4.1 Potential Mechanisms Underlying Cannabis Action on Brain Structure in Psychosis

There could be two ways in which cannabis affects brain structure in psychosis: cannabis could either directly affect brain morphology or the volumetric changes might be indirectly caused through the psychotic symptoms which are associated with cannabis use [67]. The direct mechanism could be explained as follows: two *post mortem* studies [49, 59] showed significant differences in CB1 receptor binding between schizophrenia patients and healthy controls. This suggests that changes in the endogenous cannabinoid system may be involved in the pathophysiology of schizophrenia. The endogenous cannabinoid system is fully reviewed in a separate paper published in the present issue. Dean *et al*. [59] additionally showed that acute cannabis use was associated with change in density of CB1 in tissue. Therefore, a plausible consequence of chronic cannabis use would be that these structures change in volume, which may also happen in other regions rich on CB1 receptors. However, change in receptor density due to cannabis was only shown in one *post mortem* study out of four [59]. 

It was postulated that the interaction of endogenous cannabinoids with CB1 receptors is critically involved in brain development during the sensitive period of adolescence through its regulating role in the release of glutamate. THC disturbs this normal physiological process through its action on CB1 receptors. Consequently, glutamate release is hindered which leads to neurotoxic effects and cortical structural abnormalities [13]. In contrast, two studies showed that cannabis use during adolescence was associated with more white matter compared to cannabis-naïve patients [46, 47] suggesting that cannabis-naïve individuals who develop schizophrenia might have a more vulnerable brain structure compared with that of cannabis users who develop the disease. A direct neurotoxic effect of cannabis on brain was shown by Jockers-Scherubl *et al*. [68] who reported that schizophrenia patients with regular cannabis use had significantly raised nerve growth factor serum levels compared to controls and schizophrenic patients not consuming cannabis. Additional evidence directly implicating abnormal glutamate levels in the early phases of psychosis is available from recent molecular imaging studies [69, 70].

Next to direct mechanisms, there might also be even more complex interactions between cannabis use and genetic factors that lead to brain morphologic changes, known to be involved in schizophrenia. Ho *et al*. (2011) found evidence for gene environment interactions, showing that rs12720071 genotype significantly interacts with marijuana use on white matter in schizophrenic patients. More indirectly, cannabis use was shown to be associated with poorer clinical outcome [67, 71], which in turn could also lead to a higher “toxic” effect of the psychotic state on the brain [72].

### 4.2 Limitations

Most studies included in this review had small sample sizes, leading to limited statistical power. Also, large differences in secondary variables across studies (i.e. gender, medication status, etc.) and the high overlap between cannabis and other illicit drug use may have played a confounding role. In many studies, it could therefore not explicitly be excluded that the observed effects were secondary to cannabis in contrast to other drug use. A further caveat is that there may be differences between oral ingestion and smoking cannabis; however studies did not explicitly present the methods of cannabis intake. Similar, not all studies presented mean dosage of cannabis intake prevailing any analyses on dose-response effects. For future studies we suggest including control group of cannabis-using subjects. Most studies so far have only compared cannabis using and non-using patients with cannabis naïve controls. However, this approach does not enable conclusions regarding to whether brain structural differences are caused through cannabis use or the disease of schizophrenia.

### 4.3 Conclusions

This review suggests that cannabis use in psychosis is associated with volume loss of global and specific brain structures, whereby the effects seem to be particularly strong in CB1 rich brain regions such as the cingulum, the dorsolateral prefrontal cortex and the cerebellum. As the current literature did not uncover strong similar effects in healthy samples yet, psychosis patients and subjects at risk for psychosis might be particularly vulnerable to brain volume loss due to cannabis exposure.

## Figures and Tables

**Table 1.** Overview of Structural MRI Studies Investigating Cannabis Effects

Centre	Authors and year of publication	Study design	N subjects overlapping with^a^	HC		SCZ/FEP		ARMS/GHR		Assessment of C use	Definition of C+	Definition of C-	Other substances included
				C-	C+	C-	C+	C-	C+				
Maastricht	Habets *et al*. 2011	c-s	-	48	21	28 SCZ	52 SCZ	53 GHR	33 GHR	[73]	Reported lifetime number of moderate (1-39 times) or heavy use (> 40 times) (cont)	No use	No
Iowa	Ho *et al*. 2011	c-s	-	-	-	183 SCZ	52 SCZ	-	-	CASH Interview [74]	A/D	Use but no A/D	Yes
Oxford	James *et al*. 2011	c-s	-	28	-	16 SCZ	16 SCZ	-	-	Clinical reports/ drug screening	> 3 days/week for > 6 months	No use	No
Newcastle	Cohen *et al*. 2011	c-s	-	19	17	13 FEP	6 FEP	-	.	Opiate Treatment Index [75]	NS	No use	Yes
Sydney	Solowij *et al*. 2011	c-s	-	16	15	9 SCZ	8 SCZ	-	-	Structured interview/drug screening	Daily use for 9-32 years	No use	Yes
London	Stone *et al*. 2011	c-s	-	? out of 27 ARMS	? out of 27 ARMS	-	-	? out of 27 ARMS	? out of 27 ARMS	NS	> 1 occasion in previous year (cont)	No use	Yes
Edinburgh	Welch *et al*. 2011	c-s	32 and 25 GHR, Welch *et al*. 2011	NS	NS	-	-	50 GHR	92 GHR	Self-report Face to Face Interview	Isolated, occasional or frequent use (cont)	No use	Yes
	Welch *et al*. 2011	L	-	-	-	-	-	32 GHR	25 GHR	Self-report	> 1 occasion during scan interval (2 years)	No use during scan interval	Yes
Amsterdam	Dekker *et al*. 2011	c-s	1 HC, Peters *et al*. 2009	10	-	8 SCZ	18 SCZ	-	-	Patient history	Regular use before age 15 (early onset) or regular use at age 17 or later (late onset)	No use	No
	Peters *et al*. 2009	c-s	-	21	-	11 SCZ	24 SCZ	-	-	Patient history	Use before age 17	No use before age 17	No
Utrecht	Rais *et al*. 2010	L	31 HC, 32 and 19 FEP,Rais *et al*. 2008	31	-	32 FEP	19 FEP	-	-	CIDI [73]	> 1 occasion during scan interval (5 years)	No use during scan interval	No
	Rais *et al*. 2008	L	27 FEP,Cahn *et al*. 2008	31	-	32 FEP	19 FEP	-	-	CIDI [73]	> 1 occasion during scan interval (5 years)	No use during scan interval	No
	Cahn *et al*. 2008	c-s	-	-	-	20 FEP	27 FEP	-	-	CIDI [73]	A/D	No use	No
Pittsburgh	Bangalore * et al*. 2008	c-s	-	42		24 FEP	15 FEP	-	-	SCID	Frequent or daily use (lt)	No use / once in life	No
New York	Szesko *et al*. 2007	c-s	-	56	-	31 FEP	20 FEP	-	-	SCID	A/D	No use	No

Abbreviations: A/D, DSM-IV cannabis abuse or dependence disorder; ARMS, At risk mental state; C, Cannabis; CIDI, Composite International Diagnostic Interview; cont, Cannabis as continuous variable; c-s, cross-sectional; FEP, first episode psychosis; GHR, individuals at genetic high risk for schizophrenia; HC, healthy controls; L, longitudinal; lt, lifetime; NS, not specified; SCID, Structured Clinical Interview for DSM-IV; SCZ, schizophrenia patients

a Studies are overlapping within centres

**Table 2.** Overview of *Post Mortem* Studies Investigating Cannabis Effects

Centre	Authors and year of publication	N subjects overlapping with^a^	HC		SCZ		Instrument for C use Assessment	Definition of C+	Definition of C-	Other substances included
			C-	C+	C-	C+				
Pittsburgh	Eggan *et al*. 2008	-	23	-	16	7	NS	A/D or history of cannabis use	No use	No
Sydney	Zavitsanou *et al*. 2004	-	9	-	5	5	NS	Marijuana use at some stage of subjects’ life	No use	No
	Deng *et al*. 2007	7 HC and 8 SCZ, Zavitsanou *et al*. 2004	8	-	4	4	NS	Marijuana use at some stage of subjects’ life	No use	No
Victoria	Dean *et al*. 2001	-	10	4	9	5	Toxicology/Patient history	A/D	No use	Yes

A/D, DSM-IV cannabis abuse or dependence disorder; C, Cannabis; HC, healthy controls; NS, not specified; SCZ, schizophrenia patients

**Table 3.** Brain Structural Abnormalities Revealed by MRI Studies

Centre	Authors and year of publication	Imaging Method	Image analysis	ROI / structures	Group contrasts				Main findings
					C+ vs. C- SCZ/FEP	C+ vs. C- ARMS/ GHR	C+ / C- SCZ/FEP vs. C+ / C- HC	C+ / C- ARMS/GHR vs. C+/ C-HC	
Maastricht	Habets *et al*. 2011	MRI	Voxel-based	CT	×	×	×	×	↓ CT in C+ of all groups. Significant C * group interaction
Iowa	Ho *et al*. 2011	MRI	Voxel-based	Total GM/WM, lateral ventricles	×				↓fronto-temporal WM in C+ SCZ compared to C-SCZ.
Oxford	James *et al*. 2011	DTI	Voxel-based ROI	Amygdala, hippocampus, caudate, putamen, accumbence, thalamus, pallidum	×		×		↓density in temporal fusiform gyrus, parahippocampalgyrus, ventral striatum, right middle temporal gyrus, insular cortex, precuneus, right paracingulategyrus, dorsolateral prefrontal cortex, left postcentralgyrus, lateral occipital cortex and cerebellum in C+ SCZ compared to C- SCZ. ↓FA in brain stem, internal capsule, corona radiate, superior and inferior longitudinal fasciculus in C+ SCZ compared to C- SCZ.
Newcastle	Cohen *et al*. 2011	MRI	Voxel-based	Cerebellum	×		×		No difference btw. C+ FEP and C- FEP.
Sydney	Solowij *et al*. 2011	MRI	Voxel-based	Cerebellar GM and WM	×		×		↓ cerebellar WM in C+ HC and C+ SCZ compared to C- HC and C- SCZ. No difference in WM btw. C+ HC and C+/C- SCZ.
London	Stone *et al*. 2011	MRI	Voxel-based	GM		×		×	↓ GM in prefrontal cortex associated with C in ARMS and HC. No significant group interactions.
Edinburgh	Welch *et al*. 2011	MRI	ROI	Ventricles, frontal lobe, amygdale-hippocampal complex, thalami		×		×	↑ ventricular volume associated with C in a dose-dependent manner.
	Welch *et al*. 2011	MRI	ROI	Thalami, amygdala-hippocampal complex		×			↓ bilateral thalamic volume in C+ GHR compared to C- GHR over follow up.
Amsterdam	Dekker *et al*. 2011	DTI	Voxel-based	WM, FA	×		×		↓ WM and FA in C- SCZ in the splenium of the corpus callosum compared with C+ SCZ (early onset).↓ FA in the splenium of the corpus callosum of C- SCZ. compared with C- HC.
	Peters *et al*. 2009	DTI	Voxel-based ROI	Splenium of the corpus callosum, frontal WM, parieto-occipital WM, anterior limb of internal capsule, uncinate fasciculus, arcuate fasciculus, dorsal cingulum	×		×		↑ directional coherence in the bilateral uncinate fasciculus, anterior internal capsule and frontal WM in C+ before age 17 SCZ compared to C- before age 17 SCZ.
Utrecht	Rais *et al*. 2010	MRI	Voxel-based	CT	×		×		↓ cortical thickness in DLPFC, left ACC and left occipital lobe in C+ FEP compared to C- FEP over follow up.
	Rais *et al*. 2008	MRI	Voxel-based	Total brain, GM and WM,lateral and 3^rd^ ventricle volumes	×		×		↑lateral and third ventricle volumes in C+ SCZ compared to C- SCZ and C-HC over follow up.
	Cahn *et al*. 2008	MRI		Total brain, cerebrum, cerebellum, caudate, lateral and 3^rd^ ventricle volumes	×				No difference between C+ SCZ and C- SCZ in global brain and caudate nucleus volumes.
Pittsburgh	Bangalore * et al*. 2008	MRI	Voxel-based	DLPFC,hippocampus,posterior cingulate,cerebellum	×		×		↓ GM density in right PCC in C+ FEP compared to C- FEP.
New York	Szeszko *et al*. 2007	MRI	ROI	Superior frontal gyrus, ACC, orbital frontal lobe	×		×		↓ ACC grey matter in C+ FEP compared with C- FEP and HC.

ACC, anterior cingulate cortex; ARMS, at risk mental state individuals; C, cannabis; CT, cortical thickness; DLPFC, dorsolateral prefrontal cortex; FA, fractional anisotropy; FEP, first episode psychosis patients; GHR; individuals at genetic high risk for schizophrenia; GM, gray matter; HC, healthy controls; PCC, posterior cingulate cortex; SCZ, schizophrenia patients; WM, white matter

**Table 4.** Brain Structural Abnormalities Revealed by Post Mortem Studies

Centre	Authors and year of publication	Method	Structures/receptors	Group contrasts		Findings
				C+ SCZ vs. C- SCZ	C+ / C- SCZ vs. C+ / C- HC	
Pittsburgh	Eggan *et al*. 2008	In situ hybridization and immunocytochemistry	Cortical levels of CB1RmRNA and protein	×	×	↓ levels of CB1RmRNA and protein in SCZ compared to HC but no effect of C use.
Sydney	Zavitsanou *et al*. 2004	Quantitative autoradiography	Cannabinoid CB1 receptor binding in the ACC, using the selective CB1 receptor antagonist [^3^H]SR141716A.	×	×	↑ density of CB1 receptors in SCZ compared to HC but no effect of C use.
	Deng *et al*. 2007	Quantitative autoradiography	Cannabinoid CB1 receptor binding in the superior temporal gyrus, using the selective CB1 receptor antagonist [^3^H]SR141716A and [^3^H]CP-55940.	×	×	No significant differences found in CB1 receptor density between SCZ and HC and no effect of C use.
Victoria	Dean *et al*. 2001	In situ radioligand binding and autoradiography	Cannabinoid CB1 receptor binding in the dorsolateral prefrontal cortex, using the CB1 receptor [^3^H]CP-55940.	×	×	↑ density of [^3^H]CP-55940 binding in the DLPFC in SCZ compared to HC but no effect of C diagnosis. ↑ density of [^3^H]CP-55940 binding in tissue in SCZ with THC in blood at death-

ACC, anterior cingulate cortex; C, cannabis; HC, healthy controls; SCZ, schizophrenia patients

## ABBREVIATIONS

ARMS: = At-Risk Mental State
C+: = Cannabis using group
C-: = Cannabis non-using group
FEP: = First Episode Psychosis patients
GHR: = Individuals at genetic high risk for psychosis
HC: = Healthy controls
